# The Dual Role of CCR5 in the Course of Influenza Infection: Exploring Treatment Opportunities

**DOI:** 10.3389/fimmu.2021.826621

**Published:** 2022-01-20

**Authors:** Maximiliano Ruben Ferrero, Luciana Pádua Tavares, Cristiana Couto Garcia

**Affiliations:** ^1^ Instituto de Investigación en Biomedicina de Buenos Aires (IBioBA)-CONICET-Partner Institute of the Max Planck Society, Buenos Aires, Argentina; ^2^ Laboratory of Inflammation, Oswaldo Cruz Institute, Oswaldo Cruz Foundation (FIOCRUZ), Rio de Janeiro, Brazil; ^3^ Pulmonary and Critical Care Medicine Division, Department of Medicine, Brigham and Women’s Hospital and Harvard Medical School, Boston, MA, United States; ^4^ Laboratory of Respiratory Virus and Measles, Instituto Oswaldo Cruz, FIOCRUZ, Rio de Janeiro, Brazil; ^5^ Instituto René Rachou, Fundação Oswaldo Cruz, Belo Horizonte, Brazil

**Keywords:** influenza, chemokine receptor 5, CCR5delta32, CCL5, CCL3

## Abstract

Influenza is one of the most relevant respiratory viruses to human health causing annual epidemics, and recurrent pandemics. Influenza disease is principally associated with inappropriate activation of the immune response. Chemokine receptor 5 (CCR5) and its cognate chemokines CCL3, CCL4 and CCL5 are rapidly induced upon influenza infection, contributing to leukocyte recruitment into the airways and a consequent effective antiviral response. Here we discuss the existing evidence for CCR5 role in the host immune responses to influenza virus. Complete absence of CCR5 in mice revealed the receptor’s role in coping with influenza *via* the recruitment of early memory CD8+ T cells, B cell activation and later recruitment of activated CD4+ T cells. Moreover, CCR5 contributes to inflammatory resolution by enhancing alveolar macrophages survival and reprogramming macrophages to pro-resolving phenotypes. In contrast, CCR5 activation is associated with excessive recruitment of neutrophils, inflammatory monocytes, and NK cells in models of severe influenza pneumonia. The available data suggests that, while CCL5 can play a protective role in influenza infection, CCL3 may contribute to an overwhelming inflammatory process that can harm the lung tissue. In humans, the gene encoding CCR5 might contain a 32-base pair deletion, resulting in a truncated protein. While discordant data in literature regarding this CCR5 mutation and influenza severity, the association of CCR5delta32 and HIV resistance fostered the development of different CCR5 inhibitors, now being tested in lung inflammation therapy. The potential use of CCR5 inhibitors to modulate the inflammatory response in severe human influenza infections is to be addressed.

## Introduction

Aside from the onset of Corona Virus Disease 19 (COVID-19) pandemics in 2020, influenza virus is the most relevant respiratory virus for the healthcare system, causing millions of infections worldwide annually with estimates of up to 650 thousand deaths (1, 2). Although it is unpredictable when and to which extent the circulation of influenza among humans will return to pre-COVID-19 levels, the threat is perpetual due to the high genetic variability of the virus and the existence of multiple reservoirs (3). Influenza virus belongs to the Orthomixoviridae family of segmented, negative sense, single stranded RNA viruses (4). The infection of host respiratory epithelial cells occurs through the recognition of glycoconjugates with terminal N-acetylneuraminic acid (sialic acid) in the cell membrane by the viral protein hemagglutinin (HA). The multivalent attachment to sialic acid structures triggers the endocytosis of the virus (5). Influenza A and B are the most medically relevant types among the family causing annual epidemics, whereas only Influenza A might also give rise to pandemics such as the 1918 Spanish Flu and 2009 Swine Flu, both caused by H1N1 strains subtype, that occasioned more than 50 million and 363 thousand deaths respectively (6, 7). Some influenza A avian subtypes, including H5N1 and H7N9, are highly pathogenic to humans and, although human-to-human transmission is still limited, they have been closely monitored as potential new pandemic strains (8). Despite antivirals and vaccine availability, the emergence of pandemic strains is an imminent threat due to the high genetic variability of the virus, the ability to infect birds and swine that act as reservoirs, and a decreased population immunity to new strains (9–12). Therefore, comprehending the disease mechanisms involved in respiratory virus infections and continuous viral surveillance are badly needed as they set the basis for new therapeutics.

Dysfunctional inflammation triggered by influenza infection is related to the clinical manifestations and is orchestrated by different mediators (e.g: leukotrienes, cytokines and chemokines) and cell types (e.g: leukocytes, epithelial and endothelial cells) (13). However, a regulated well-controlled response ensures a proper viral clearance with restoration to tissue homeostasis. Therefore, inflammation has a dual role during influenza infection and disease. Although the chemokine receptor CCR5 does not actively participate in the infection process of influenza, after its activation by the chemokines CCL3/MIP-1α, CCL4/MIP-1β, and CCL5/RANTES, it becomes a key player in the inflammatory milieu that contributes to infection restraint. However, it might also be associated with inflammatory bystander lung damage. Here we discuss this duality of CCR5 activation during influenza infection.

## Expression of CCR5 and CCR5 Ligands Upon Influenza Infection

One of the first reactions of the host after influenza infection is the production of CCR5 ligands by lung resident cells, especially alveolar macrophages and epithelial cells (14–16). CCL5 can be detected in human bronchoalveolar lavage fluid (BALF) samples after 7 days of symptoms onset (17). *In vitro* infection of type 2 human alveolar epithelial cells (hAECII) with either H1N1 or H5N1 virus leads to a significant production of CCL5 by those cells, showing that they may be a principal source of CCL5 during influenza pneumonia. In addition, human alveolar macrophages exposed to both H1N1 and H5N1 viruses produced CCL5. Interestingly, the infection with the H5N1 virus, a more pathogenic subtype, led to stronger CCL5 production in both cell types (15, 18). CCL3 and CCL4, the other CCR5 ligands, are also expressed in response to experimental influenza infection in human volunteers (19). A study of over 15 critically ill patients showed that CCL3 is augmented in lung aspirates of patients, and notably, at the serum level, there was an increment of CCL3 and CCL4 in comparison with mild cases of influenza infection (20). In addition, there is recent evidence showing, at the mRNA level, that peripheral blood monocytes derived from hospitalized patients diagnosed with influenza A or Severe Acute Respiratory Syndrome Virus-2(SARS-CoV-2) infection overexpress CCL3 (21).

In murine models, all CCR5 ligands are produced in lung tissue in response to influenza infection (22, 23). This contributes to the acute recruitment of leukocytes from the innate immunity to the lungs, mainly inflammatory monocytes and neutrophils, but also NK cells, which can induce CCR5 expression in response to the infection (24, 25). The latter recruitment of cells from adaptive immunity is also mediated by CCR5. Indeed, effector cytotoxic Th1 lymphocytes, memory CD8 T cells, and also B lymphocytes express CCR5. Moreover, there is evidence pointing that CCL5:CCR5 interaction contributes to the formation of inducible bronchus-associated lymphoid tissue iBALT in mice (14, 23, 26).

## The Role for CCR5 in Inflammation and Immunity to Influenza Virus

**Figure 1:**
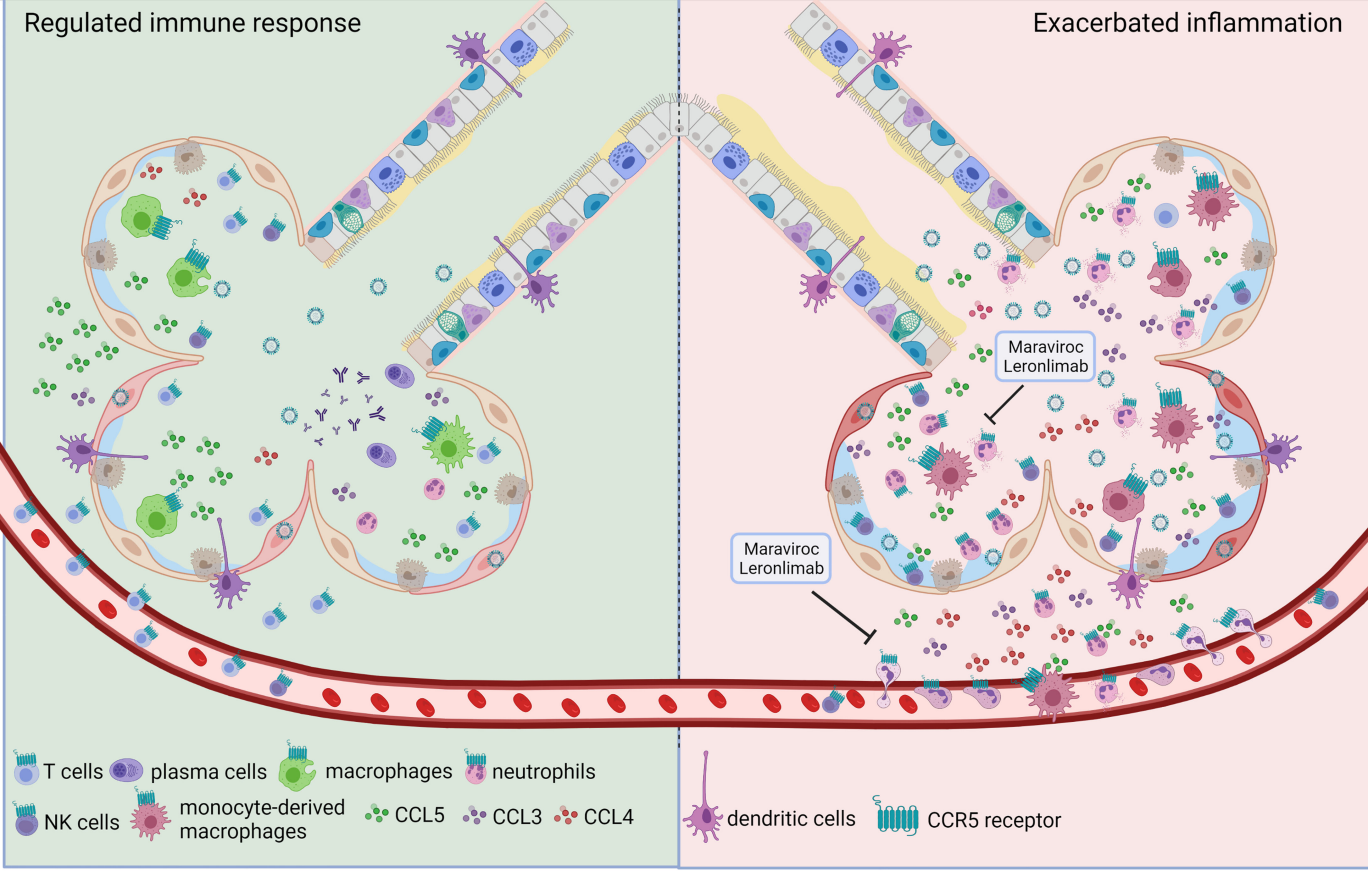
The dual role of CCR5 during influenza infection. Triggered by influenza infection, one of the first reactions of epithelial cells and resident alveolar macrophages is the production of CCR5 ligands. CCL5:CCR5 interaction is necessary for the development of a proper immune response (left side) to restrain viral expansion since it favors resident macrophages survival, promotes reprogramming of macrophages to pro-resolving phenotypes, mediates the recruitment of T lymphocytes and the establishment of iBALT contributing to immunological memory. However, uncontrolled activation of many components of the immune system after influenza infection is associated with severe pulmonary damage (right side). In this scenario, increased recruitment of neutrophils, inflammatory monocytes and natural killer cells can be mediated by CCR5 expression on those cells, and the actual evidence shows that CCL3 may be related to this exacerbated response. In this situation CCR5 inhibition by Maraviroc or Leronlimab, might represent an interesting therapeutic alternative.

The immune responses that follow influenza infection are crucial to control virus proliferation and for the development of memory responses; however, uncontrolled, or exaggerated activation of the many components of the immune system is associated with severe pulmonary damage and contributes to flu mortality (13). Thus, the immune responses must be finely regulated and coordinated to ensure viral clearance and restoration of lung homeostasis, with minimum bystander damage. As part of the host immune circuits for resistance to infection, CCR5 mediates the recruitment and activation of leukocytes during influenza. Interestingly, CCR5 plays contrasting roles in different inflammatory and infectious diseases leading to protection against certain pathogens or immunopathology triggered by exacerbated inflammation (27, 28). In this regard, CCR5 activation during different phases of influenza infection might also lead to different outcomes. Indeed, CCR5 activation during the initial stages of influenza infection ensure the proper recruitment of leukocytes and activation of antiviral pathways in the epithelial cells (**Figure 1** left panel) (14, 23, 29, 30). However, sustained or exaggerated CCR5 activation during severe/exacerbated influenza infection might fuel the inflammatory responses leading to increase pulmonary damage and dysfunction (**Figure 1** right panel). The cellular expression of CCR5 dictates what cell type can be recruited by mediators such as CCL3, CCL4 and CCL5, the classical CCR5-associated chemokines.

CCR5 and its cognate chemokines are rapidly induced post-influenza infection in both humand and mice and ensure the prompt recruitment of leukocytes to the airways for an effective response (31). Indeed, the development and use of CCR5 knockout mice shed light on the mechanistic role for CCR5 mediating host protection to influenza. CCR5 deficient mice are highly susceptible to influenza infections and present increased neutrophilic inflammation and lung dysfunction in comparison to wild type mice (23). During influenza infection in mice, neutrophil expression of CCR5 is significantly increased and promotes different ex vivo cell functions (25). Whether CCR5 signaling *in vivo* directly regulates neutrophil activation or recruitment during influenza is yet to be explored; nevertheless, CCL5:CCR5 was shown to promote reprogramming of murine macrophages to pro-resolving phenotypes contributing to resolution of inflammation (32). In addition, CCR5:CCL5 was shown to prevent virus-induced apoptosis of human and mouse macrophages during influenza infection (33). Alveolar macrophages are crucial cells for viral and apoptotic cell clearance during infections preventing further unnecessary inflammatory responses in the lungs and tissue damage (34). Therefore, CCR5 signaling aids to the regulation of macrophage regulatory responses to guarantee restoration of tissue homeostasis during influenza infections.

NK cells are also recruited by CCR5 (24) and play a role in immunity to influenza infections in humans and mice (35, 36). NK cells can interact with influenza-infected cells and with the virus itself leading to secretion of cytokines and cytotoxic granules that restrain viral replication within the early stages of infection (36, 37). On the other hand, influenza virus can directly impair NK function to evade this innate layer of host immunity (38) and exaggerated NK cell activation, rather than being protective, might contribute to lung damage during severe influenza infections (39). Whether CCR5 activation transduces a protective or pathological NK cell response during influenza is yet to be determined. In parallel, the recruitment and activation of γδ T cells, mainly by CCR5, are important components of the potent antiviral responses to influenza infection in humans (40, 41).

In addition to the above mentioned role in innate immunity to influenza, CCR5 signaling is also necessary for the recruitment and effective response of the components of the adaptive immune system (42). Indeed, the increased pathology and lung dysfunction of CCR5 deficient mice are associated with decreased recruitment of CD8+ T cells during infection (23). Moreover, CCR5 was shown to be important for the development of early CD8+ T cell memory leading to control of virus replication during a secondary infectious challenge in mice (14). Furthermore, CCR5 might also impact B cell activation and recruitment during influenza. Secretion of CCL3 and CCL4 by B cells can lead to the recruitment of activated follicular CCR5+ CD4+ T cells, which enhances interaction between these two cell types and improves humoral immunity (43). Akin with that, mice lacking the CCL5 scavenger Atypical Chemokine Receptor 2 (ACKR2) present increased CCL5 levels, CCR5+CD4+ lymphocyte recruitment to the airways and augmented levels of IgA in the BALF during influenza. The specific phenotype of the T CD4+ recruited *via* CCR5 during influenza is yet to be defined (23). Noteworthy, the antagonism of CCR5 using maraviroc has not impaired the humoral response of HIV patients to the 2009 pandemic influenza A-H1N1 adjuvanted vaccine (44).

Pulmonary epithelial cells, in addition to the leukocytes, are important players driving antiviral responses to influenza (29). More recently, the direct role of CCL5:CCR5 in epithelial antiviral responses was uncovered. CCL5:CCR5 was shown to reduce influenza A replication in human epithelial cells by inducting the antiviral restriction factor SAM domain and HD domain-containing protein 1 (SAMHD1) (30). Keeping with that, the CCR5 agonist gp120 was shown to reduce A(H1N1)pdm09 replication *in vitro* in an IFITM3-dependent manner in human macrophages and human epithelial cervical cancer (HeLa) cells (45). Therefore, CCR5 signaling can impact the antiviral responses mediated by both epithelial and immune cells and, this should be taken into consideration when developing therapeutic strategies targeting this receptor for other inflammatory diseases. Interestingly, a recent study provided strong evidence for the protective role of CCR5 antagonism during Chronic Obstructive Pulmonary Disease (COPD) exacerbations caused by influenza in which CCL3 levels but no CCL5, correlated with an exacerbated inflammatory process (46). Maraviroc treatment during COPD viral exacerbations protected mice from the lethal pulmonary inflammation without affecting viral replication (46). In this regard, understanding the response to the virus and distinguishing between harmful and protective inflammation is crucial. The most relevant findings regarding CCR5 role during influenza infection are summarized on **Table 1**.

**Table 1:** Evidence over CCR5 role on the immune response to influenza virus.

Strategy	Influenza strain	Model	Findings
CCR5 Knockout	A/WS/SS H1N1	Mouse	CCR5 KO and CCL5 KO have higher mortality and increased apoptosis of macrophages at day 9 post-infection (33)
Anti-CCR5 specific antibody	A/WS/SS H1N1	Human macrophages	CCR5 blockage increases the proportion of apoptotic macrophages post-influenza infection *in vitro* (33)
CCR5 knockout	A/HK-x31 H3N2 and A/Puerto Rico/8/1934 H1N1	Mouse	CCR5 knockout mice have impaired induction of T CD8^+^ memory cells post-influenza infection and increased viral titers in a secondary viral challenge (14).
Maraviroc	2009 pandemic influenza AH1N1v	HIV patients	Pharmacological blockage of CCR5 does not impact antibody responses triggered by vaccination (44).
CCR5 knockout	A/Puerto Rico/8/1934 H1N1	Mouse	CCR5 knockout mice have diminished numbers of NK cells in the bone marrow, post-infection (35).
HIV glycoprotein gp120	A(H1N1)pdm09	Human epithelial cervical cancer (HeLa) cells	Gp120 acts as an agonist for CCR5 and inhibits influenza replication in HeLa cells (45).
CCR5 Knockout	A/WSN/33 H1N1	Mouse	CCR5 KO mice present increased pulmonary neutrophilic inflammation and damage, and reduced T CD8^+^ lymphocyte recruitment during influenza infection. (23)
CCR5 agonism (CCL5)	A/Switzerland/9715293/2013 H3N2	Human epithelial cell line (A549)	CCL5 binding to CCR5 increases SAMHD1 and prevents viral replication and epithelial cell death *in vitro* (30).
Maraviroc	A/Puerto Rico/8/1934 H1N1	Mouse model of influenza-induced COPD exacerbation	Pharmacological blockage of CCR5 reduced lethality, neutrophilic inflammation, pulmonary damage without affecting viral titers (46).

## CCR5delta32 and Disease Severity

The gene encoding CCR5 might contain a 32 base pair deletion within the exon 3 resulting in a truncated protein that cannot be expressed on cell surface and therefore is non-functional (47). This deletion is present at different frequencies on populations around the world, which is related to ancestry. Whereas the allele frequency (AF) of the deletion is more than 15% in some European countries like Norway, Estonia and Latvia, some Asian and African countries present CCR5delta32 AF lower than 1% (48). Delta32 deletion was discovered in individuals multiply-exposed to HIV that were resistant to the infection and carried two alleles of CCR5-delta32 (49). This resistance was observed in CCR5-tropic HIV strains which depend on CCR5 as a co-receptor for cell entry. This process is avoided when a non-functional CCR5 is present in every cell on CCR5-delta32 homozygousity and act as a dominant-negative on the expression of wild type CCR5 and also C-X-C Motif Chemokine Receptor 4 (CXCR4), the other co-receptor of HIV (50).

While CCR5-delta32 homozygosis confers protection to HIV, meta-analysis have shown that HIV susceptibility or perinatal infection are not affected by CCR5-delta32 heterozygosity (51–53). Upon this findings on HIV resistance, CCR5 blockers or antagonists started to be tested against Acquired Immune Deficiency Syndrome (AIDS) and currently the CCR5 antagonist Maraviroc is clinically used (54). Moreover, a patient with acute myeloid leukemia and HIV had the infection controlled by the transplant of stem cells from a homozygous delta32 donor (55).

Besides HIV, CCR5delta32 has been associated with susceptibility (56, 57) or protection to different diseases, including COVID-19 (58–60). Regarding influenza, discordant data are present in literature. After the 2009 H1N1 pandemics, studies on distinct populations evaluated the CCR5delta32 allele frequencies on influenza patients with different outcomes. The first published study, assessing only 20 cases in Canada, found that the CCR5delta32 was a risk factor for the severity of H1N1 infection in white patients (61). In 2013 one Spanish study comparing a mild and a fatal case of the pandemic H1N1 infection found that the fatal case was homozygous for the CCR5Δ32 allele (62). Another Spanish study from 2015, assessing a larger population of 171 influenza patients found a correlation of CCR5Δ32 and mortality (63). On the other hand, three studies, one with 29 European (mostly Italian), other with 330 Brazilians and another with 432 Brazilian influenza patients with different clinical manifestations found no association between CCR5Δ32 and H1N1 severity (64–66). The conflict data might be explained by the global distribution of CCR5Δ32 allele. CCR5delta32 AF in countries where associations with influenza outcomes were found – Canada and Spain – are higher (8.1% and 7%, respectively) than in countries where no association was found – Italy (6.27%) and Brazil (4-5.44%) (48, 67).

## CCR5 as Potential Target to Modulate Inflammation in Lung

Severe pneumonia following viral infection is principally associated with an overwhelmed production of inflammatory mediators and leukocyte recruitment to lung tissue. For that reason, chemokine receptors are interesting therapeutic candidates for lung inflammation. As aforementioned, CCR5 contribution during influenza infection appears to be crucial for the development of an antiviral response and the proper induction of immunologic memory. On the other hand, CCR5 activation is associated with excessive recruitment of neutrophils, inflammatory monocytes and NK cells in models of severe influenza pneumonia (24, 46, 68). This dual role of a chemokine receptor in the context of lung diseases is not an exclusive characteristic of CCR5 (69). Currently, the information obtained by the use of animal models suggests that while some CCR5 ligands, like CCL5, can play a protective role in influenza infection (23, 33) others, like CCL3, may contribute to an overwhelming inflammatory process which can harm the lung tissue (46, 70). Many pharmacological strategies that aim to impair CCR5 activity and endocytosis have been developed to fight HIV infection and were already tested in humans showing good safety profiles and effective antagonism properties. Nowadays, repositioning strategies based on the well-established CCR5-inhibitory capacities of drugs like Maraviroc, the only CCR5 inhibitor approved for clinical use, and Leronlimab, a CCR5-specific human IgG4 monoclonal antibody, succeed at presenting a good anti-inflammatory performance in the context of lung inflammatory conditions. Indeed, it was recently published that Leronlimab treatment reduced plasma IL-6 and viral load in critical COVID-19 patients (71). Besides, new CCR5 antagonists like cenicriviroc, which also present CCR2 inhibition, and GRL-117C, arise opportunities for the discovery of novel anti-inflammatory treatments focusing on CCR5 in the near future (72, 73). Currently, there is no disclosed clinical trial attempting to assess whether CCR5 antagonism can improve patient outcome during severe influenza pneumonia. However, as the current COVID-19 pandemics brought up challenging times while also emphazised a pre-existing demand for novel treatments to control the inflammatory response in the lungs, at least five clinical trials are being conducted to study CCR5 as potential drug target to treat lung inflammation during SARS-CoV-2 infection (NCT04441385, NCT04475991, NCT04710199, NCT04901676 NCT04901689). Either by CCR5 antagonism with Maraviroc or by its blockage with Leronlimab, these trials attempt to control the excessive inflammatory response by decreasing leukocyte accumulation in the lungs and inflammatory mediators in plasma of COVID-19 patients which is expected to improve patients outcome. By the moment, four of these clinical trials are on recruiting phase and the only completed study has no posted results yet (NCT04710199).

## Conclusions

CCR5 plays important roles during influenza infection (**Figure 1**) by contributing to a suitable immune response *via* CCL5 to cope with the viral infection, but also subsidizing excessive inflammation and tissue damage by mechanisms associated with increased CCL3 production. This ambivalent character of CCR5 on influenza infection is not unique to this chemokine receptor but observed for many others in the dispute between pathological lung inflammation and restoration of physiological state. Thus, the correct use of CCR5 inhibitors as potential anti-inflammatory drugs in severe influenza infections requires a profound knowledge of the different phases in the inflammatory processes to be modulated.

## Author Contributions

MF, LT, and CG contributed to the conception and design of the study; wrote the manuscript and discussed the content. All authors contributed to the article and approved the submitted version.

## Funding

This work was supported by Fundação Oswaldo Cruz/Fiocruz.

## Conflict of Interest

The authors declare that the research was conducted in the absence of any commercial or financial relationships that could be construed as a potential conflict of interest.

## Publisher’s Note

All claims expressed in this article are solely those of the authors and do not necessarily represent those of their affiliated organizations, or those of the publisher, the editors and the reviewers. Any product that may be evaluated in this article, or claim that may be made by its manufacturer, is not guaranteed or endorsed by the publisher.

## Abbreviations

CCR5, Chemokine receptor 5; CCL3, C-C Motif Chemokine Ligand 3; CCL4, C-C Motif Chemokine Ligand 4; CCL5, C-C Motif Chemokine Ligand 5; COVID-19, Corona Virus Disease 19; BALF, the bronchoalveolar lavage fluid; hAECII, human alveolar epithelial cells; SARS-CoV-2, Severe Acute Respiratory Syndrome Virus-2; NK, Natural Killer; ACKR2, Atypical Chemokine Receptor 2; HIV, Human immunodeficiency virus; SAMHD1, SAM domain and HD domain-containing protein 1; COPD, chronic obstructive pulmonary disease, AF, allele frequency, CXCR4, C-X-C Motif Chemokine Receptor 4; AIDS, Acquired Immune Deficiency Syndrome.
